# A retrospective study of sepsis-associated encephalopathy: epidemiology, clinical features and adverse outcomes

**DOI:** 10.1186/s12873-020-00374-3

**Published:** 2020-10-06

**Authors:** Jiayi Chen, Xiaobei Shi, Mengyuan Diao, Guangyong Jin, Ying Zhu, Wei Hu, Shaosong Xi

**Affiliations:** 1 Department of Critical Care Medicine, Affiliated Hangzhou First People’s Hospital, Zhejiang University School of Medicine, No.261, Huansha Road, Zhejiang, 310006 Hangzhou China; 2 Department of Radiology, Affiliated Hangzhou First People’s Hospital, Zhejiang University School of Medicine, Zhejiang, Hangzhou China

**Keywords:** Sepsis, Sepsis-associated encephalopathy, Retrospective study

## Abstract

**Background:**

Sepsis-associated encephalopathy (SAE) is a common complication of sepsis that may result in worse outcomes. This study was designed to determine the epidemiology, clinical features, and risk factors of SAE.

**Methods:**

This was a retrospective study of all patients with sepsis who were admitted to the Critical Care Medicine Department of Hangzhou First People’s Hospital Affiliated with Zhejiang University School of Medicine from January 2015 to December 2019.

**Results:**

A total of 291 sepsis patients were screened, and 127 (43.6%) were diagnosed with SAE. There were significant differences in median age, proportion of underlying diseases such as hypertension, Sequential Organ Failure Assessment (SOFA) score, Acute Physiology and Chronic Health Evaluation II (APACHE II) score, gastrointestinal infections, detection rate of *Enterococcus*, and 28-day mortality between the SAE and non-SAE groups. Both the SOFA score and APACHE II score were independent risk factors for SAE in patients with sepsis. All 127 SAE patients were divided into survival and non-survival groups. The age, SOFA score, and APACHE II score were independently associated with 28-day mortality in SAE patients.

**Conclusion:**

In the present retrospective study, nearly half of patients with sepsis developed SAE, which was closely related to poor outcomes. Both the SOFA score and APACHE II score were independent risk factors for predicting the occurrence and adverse outcome of SAE.

## Background

Sepsis-associated encephalopathy (SAE) is a common complication of patients with sepsis and can manifest as mild disturbance of consciousness, disorientation, cognitive impairment, convulsion or deep coma [1, 2]. Importantly, SAE can result in dramatically poorer outcomes in patients with sepsis, with the mortality increasing with a rising SAE severity to a maximum of 70% [3]. Surviving SAE patients are likely to exhibit prolonged or permanent side effects, including altered behaviour, cognitive impairment, reduced quality of life, or premature death [4]. To date, the diagnostic criteria and potential risk factors for SAE remain incompletely understood. Early identification, timely diagnosis and effective management may be important for the disease control in sepsis patients. The present study was therefore designed to explore the epidemiology, clinical characteristics and risk factors for SAE, as well as its adverse outcomes, via a retrospective study of 291 patients with sepsis.

## Methods

### Patient selection

This was a retrospective study of all patients with sepsis who were admitted to the Critical Care Medicine Department of Hangzhou First People’s Hospital Affiliated with Zhejiang University School of Medicine from January 2015 to December 2019. Patient inclusion criteria were as follows: (1) Diagnosis of sepsis: Patients were diagnosed with sepsis based upon the sepsis 3.0 definition, which is the life-threatening organ dysfunction caused by the host’s maladjusted response to infection [5]. (2) SAE identification: Cognitive and neuropsychiatric disorders clearly documented by medical staff (doctors and nurses), as well as a Glasgow coma score (GCS) < 15 or manifestations of delirium (including inattention, disorientation, altered thinking, decreased psychomotor activity, and/or agitation) confirmed by the Confusion Assessment Method for the Intensive Care Unit (CAM-ICU). Patient exclusion criteria were sedative-related cognitive effects, primary central nervous system disease (cerebral vascular disease, central nervous system infection, autoimmune encephalitis, seizures), metabolic encephalopathy (hypoglycaemia, diabetic ketoacidosis, hepatic encephalopathy, pulmonary encephalopathy, uraemic encephalopathy), and toxicosis.

### Data collection

For each patient included in this study, we collected the following data at the onset of sepsis or SAE: (1) General data: including age, gender, underlying disease; (2) Data at ICU admission: Acute Physiology and Chronic Health Evaluation II (APACHE II) score, Sequential Organ Failure Assessment score (SOFA score), site of infection, haematological findings (white blood cell (WBC) count, platelet (PLT) count, haematocrit (HCT), procalcitonin (PCT), and serum creatinine (Cr) levels), aetiological information, and outcome indexes (length of stay in the hospital, 28-day mortality).

### Assessment of risk of bias

Two authors independently assessed the risk of bias for each case using the following information outlined: whether the identification of each case of sepsis or SAE was the joint decision of more than three members of the research team; and whether there was evidence of selective reporting of outcomes. Any disagreement was resolved by discussion or by involving a third assessor.

### Statistical analysis

All data were analysed using SPSS 22.0 (SPSS, Inc., NY, USA). Categorical and continuous variables are given as numbers (percentages) and medians [25th–75th percentiles], respectively, and were compared via Mann-Whitney U tests, χ2 tests, or Fisher’s exact tests, as appropriate. SAE- and survival-associated risk factors were identified via multivariate logistic regression analysis. Kaplan-Meier curves were analysed by the log-rank test. *P* <  0.05 was the significance threshold.

## Results

### Baseline characteristics

**Fig. 1:**
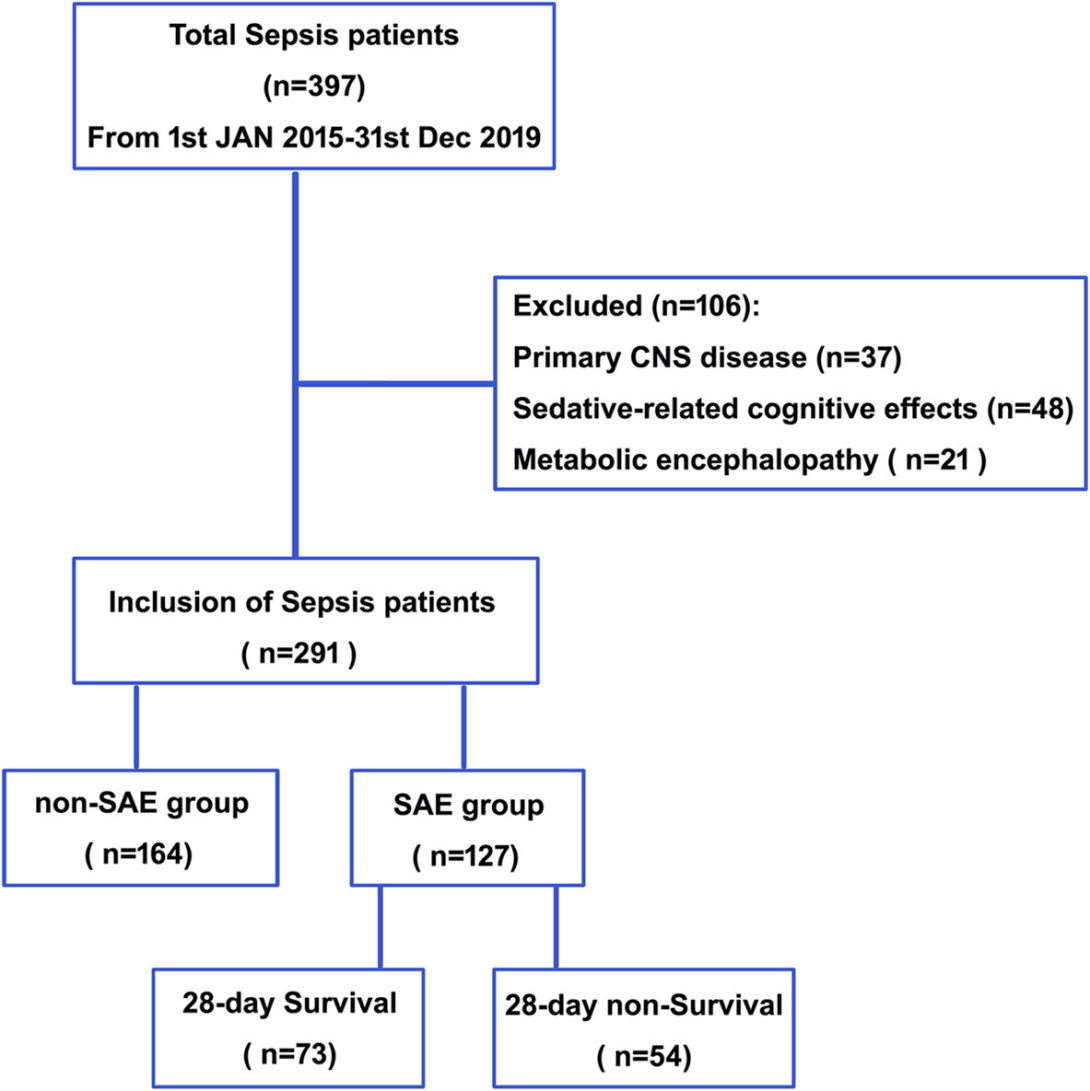
Flowchart of patient cohorts. SAE = sepsis-associated encephalopathy. CNS = central nervous system disease

After excluding 106 patients, a total of 291 patients with sepsis were retrospectively screened and assigned to the SAE group and non-SAE group. According to the outcome of 28-day mortality, the SAE patients were stratified into survival and non-survival groups (Fig. 1).

Of these 291 sepsis patients, 127 (43.6%; 90 males) were diagnosed with SAE. The median ages of the SAE and non-SAE groups were 64 [48, 76] and 55[43, 68] years old, respectively, with SAE patients being significantly older than non-SAE patients (*P* <  0.01). We also found significant differences between these two groups with respect to the SOFA score, APACHE II score, and proportion of underlying diseases such as hypertension (*P* <  0.05; Table 1).

**Table 1:** Patients’ baseline characteristics

	All patients ***n*** = 291	SAE ***n*** = 127	non-SAE ***n*** = 164	***P***
**Age** (years), median	59 [45, 72]	64 [48, 76]	55 [43, 68]	< 0.01*
**Sex** (%)				
Male	213 (73.2)	90 (70.9)	123 (75.0)	0.430
Female	78 (26.8)	37 (29.1)	41 (25.0)	
**Underlying diseases** (%)				
Hypertension	75 (25.8)	41 (32.3)	34 (20.7)	0.025*
Diabetes	40 (13.7)	23 (18.1)	17 (10.4)	0.057
COPD	28 (9.6)	10 (7.9)	18 (11.0)	0.374
CHF	37 (12.7)	19 (15.0)	18 (11.0)	0.312
CHD	12 (4.1)	4 (3.1)	8 (4.9)	0.462
CKD	17 (5.8)	7 (5.5)	10 (6.1)	0.833
Malignant tumor	44 (15.1)	22 (17.3)	22 (13.4)	0.356
Immune diseases	4 (1.4)	1 (0.8)	3 (1.8)	0.803
Others	26 (8.9)	13 (10.2)	13 (7.9)	0.493
**Disease severity** (median)				
SOFA score	5 [2, 7]	7 [5, 10]	3 [1, 5]	< 0.01*
APACHE II score	11 [8, 15]	15 [12, 19.5]	9 [6, 11]	< 0.01*
**Outcome**				
Hospital LOS (median)	20 [11, 30]	19 [9, 28]	21 [13, 36]	0.143
28-day mortality (%)	77 (26.5)	54 (42.5)	21 (12.8)	< 0.01**

*SAE* sepsis-associated encephalopathy, *COPD* chronic obstructive pulmonary disease, *CHF* chronic heart failure, *CHD* chronic hepatic disease, *CKD* chronic kidney disease, *SOFA* sequential organ failure assessment, *APACHE II* acute physiology, age and chronic health evaluation II, *LOS* length of stay

*Statistical analysis using Mann Whitney test, **Statistical analysis using Fisher’s exact probability method

The primary outcome measures that were monitored in the present study included length of stay (LOS) in the hospital and 28-day mortality. The results showed no significant differences in hospital LOS between the SAE and non-SAE groups. However, we found that the 28-day mortality of SAE patients was significantly higher than that of non-SAE patients (42.5% vs. 12.8%, *P* <  0.01; Table 1).

**Fig. 2:**
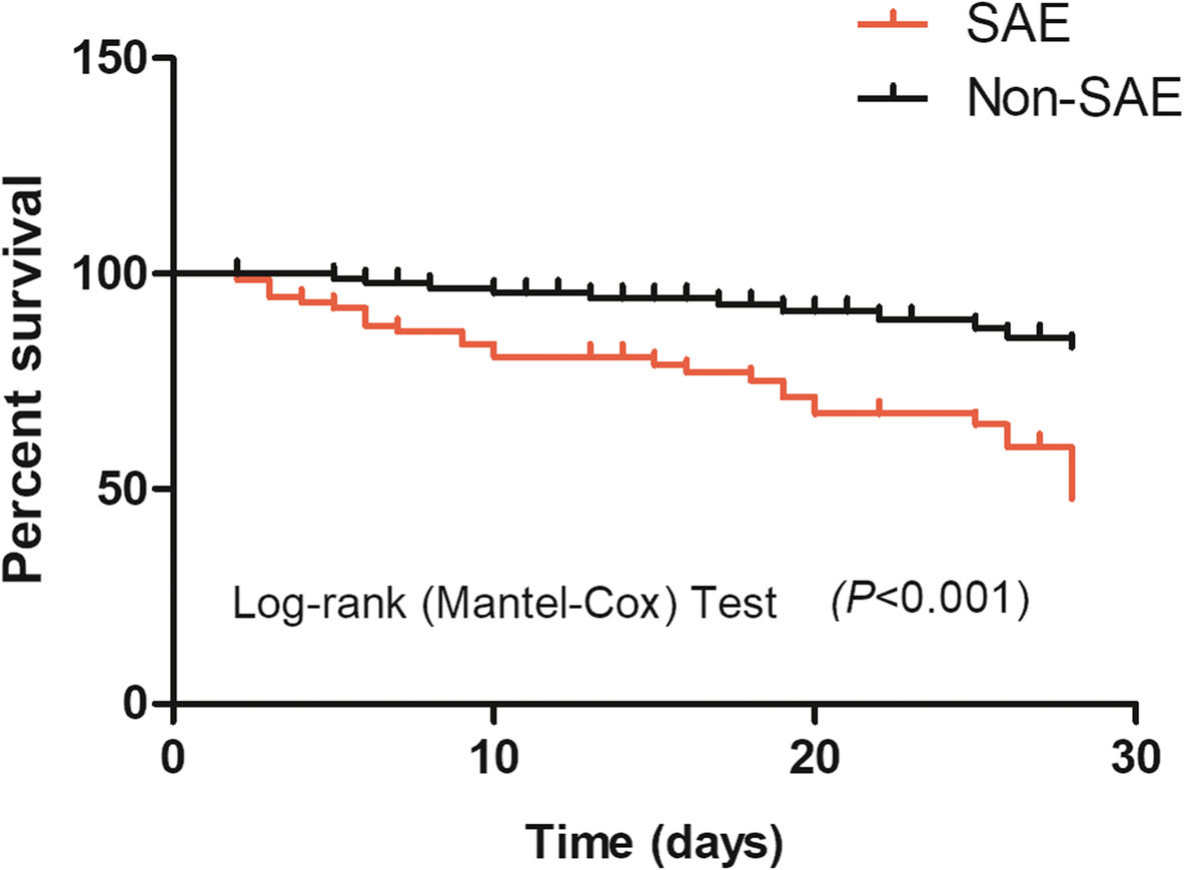
Kaplan-Meier survival analysis of sepsis patients over 28 days. The SAE group had a significantly poorer 28-day survival than non-SAE group (HR = 3.890, 95%CI: 2.035 ~ 7.437; *P* < 0.001). SAE = sepsis-associated encephalopathy

A Kaplan-Meier survival analysis further confirmed that an SAE diagnosis was associated with significantly poorer 28-day survival in sepsis patients (HR = 3.890, 95% CI: 2.035–7.437; *P* <  0.001; Fig. 2).

### Comparison of clinical and etiological findings between SAE and non-SAE patients

A total of 291 patients with sepsis were admitted to the hospital due to medical disease, emergency surgery or elective surgery. There were no significant differences in the disease types of the patients (*P* = 0.358). The main infection sites were the respiratory tract, biliary tract, gastrointestinal tract, urinary tract, bloodstream, skin and soft tissue. The main pathogens detected were *Staphylococcus*, *Escherichia coli*, *Enterococcus*, *Acinetobacter baumannii*, *Pseudomonas aeruginosa*, *Klebsiella pneumoniae*, and fungi (*Candida*). We found that patients in the SAE group had significantly higher rates of gastrointestinal infections than non-SAE patients (26.8% vs. 15.2%, *P* = 0.015). In addition, these SAE patients had a higher rate of detection of *Enterococcus* than non-SAE patients (16.5% vs. 7.9%, *P* = 0.023). No significant differences were observed between groups with respect to other pathogens or infection sites (*P* > 0.05; Table 2). Furthermore, these SAE patients had higher serum PCT concentrations (2.33 [1.14, 6.97] vs. 1.39 [0.42, 3.99], *P* <  0.01) and lower platelet counts (105 [56.0, 180.0] vs. 168 [110.0, 246.5], *P* < 0.01) than did their non-SAE counterparts (Table 2).

**Table 2:** Comparison of clinical and etiological findings in sepsis patients

	All patients n = 291	SAE n = 127	non-SAE n = 164	***P***
**Disease type** *n* (%)				
Medical disease	198 (68.0)	88 (69.3)	110 (67.1)	0.358
Emergency surgery	47 (16.2)	23 (18.1)	24 (14.6)	
Elective surgery	46 (15.8)	16 (12.6)	30 (18.3)	
**Infection sites** *n* (%)				
Respiratory tract	159 (54.6)	76 (59.8)	83 (50.6)	0.076
Biliary tract	25 (8.6)	8 (6.3)	17 (10.4)	0.220
Gastrointestinal tract	59 (20.3)	34 (26.8)	25 (15.2)	0.015**
Urinary tract	16 (5.5)	9 (7.1)	7 (4.3)	0.296
Bloodstream	14 (4.8)	7 (5.5)	7 (4.3)	0.623
Skin and soft tissue	15 (5.2)	5 (3.9)	10 (6.1)	0.408
**Detected pathogens** *n* (%)				
Staphylococcus	35 (12.3)	16 (12.6)	19 (11.6)	0.792
Enterococcus	34 (15.5)	21 (16.5)	13 (7.9)	0.023*
*Escherichia coli*	67 (30.6)	31 (24.4)	36 (21.9)	0.621
Acinetobacter	61 (20.9)	27 (21.3)	34 (20.7)	0.913
Pseudomonas	18 (6.2)	8 (6.3)	10 (6.1)	0.944
Klebsiella	27 (9.3)	11 (8.7)	16 (9.8)	0.750
Fungus	32 (10.9)	13 (10.2)	19 (11.6)	0.715
Others	15 (5.2)	6 (4.7)	9 (5.5)	0.770
**Biochemical Indexes** (median)				
PCT (μg/L)	1.89 [0.66,5.37]	2.33 [1.14, 6.97]	1.39 [0.42, 3.99]	< 0.01*
Endotoxin (EU/mL)	0.11 [0.05, 0.20]	0.12 [0.05, 0.21]	0.10 [0.05, 0.19]	0.464
WBC (×10^9^/L)	10.8 [7.60, 15.8]	10.9 [8.5, 16.2]	11.35 [7.70, 15.88]	0.245
PLT (×10^9^/L)	152 [76.7, 191.5]	105 [56.0, 180.0]	168 [110.0, 246.5]	< 0.01*
HCT	30.1 [27.0, 33.0]	20.3 [18.1, 22.1]	33.4 [33.0, 34.3]	0.069
Cr (μmol/L)	78 [52.0, 95.8]	78 [59.0, 109.8]	73.5 [51.5, 87.0]	0.315

*SAE* sepsis-associated encephalopathy, *PCT* procalcitonin, *WBC* white blood cell, *PLT* blood platelet, *HCT* hematocrit, *Cr* serum creatinine

*Statistical analysis using Mann Whitney test, **Statistical analysis using Fisher’s exact probability method

### Multivariate analysis of SAE risk factors in sepsis patients

Based on the above bivariate analysis, after adjusting the baseline characteristics, infection site, patient type, clinical and aetiological findings of sepsis patients, a subsequent multivariate analysis revealed that both SOFA score (OR per 1-score increment: 1.421, 95% CI: 1.244–1.623, *P* < 0.001) and APACHE II score (OR per 1-score increment: 1.239, 95% CI: 1.144–1.341, *P* < 0.001) were independent risk factors for SAE in ICU patients with sepsis (Table 3).

**Table 3:** Multivariate analysis of risk factors in Sepsis-Associated Encephalopathy patients

Value	OR	95% CI	***P***
APACHE II (per 1-score increment)	1.239	1.144 ~ 1.341	< 0.001
SOFA (per 1-score increment)	1.421	1.244 ~ 1.623	< 0.001

*SOFA* sequential organ failure assessment, *APACHE II* acute physiology, age and chronic health evaluation II, *LOS* length of stay

### Multivariate analysis of poor outcome-related factors in SAE patients

Based upon the outcome of 28-day mortality, 127 SAE patients were divided into a survival group and a non-survival group. A multivariate analysis revealed that age (OR per 1-year increment: 1.059, 95% CI: 1.027 ~ 1.093, *P* < 0.001), SOFA score (OR per 1-score increment: 1.167, 95% CI: 1.009 ~ 1.349, *P* = 0.037), and APACHE II score (OR per 1-score increment: 1.178, 95% CI: 1.063 ~ 1.305, *P* < 0.01) were independently associated with 28-day mortality in SAE patients (Table 4).

**Table 4:** Multivariate analysis of 28-day mortality in Sepsis-Associated Encephalopathy patients

Value	OR	95% CI	***P***
Age (per 1-year increment)	1.059	1.027 ~ 1.093	< 0.001
SOFA score (per 1-score increment)	1.167	1.009 ~ 1.349	0.037
APACHE II score (per 1-score increment)	1.178	1.063 ~ 1.305	< 0.01

*SOFA* sequential organ failure assessment, *APACHE II* acute physiology, age and chronic health evaluation II

## Discussion

The occurrence of SAE is one of the main manifestations of organ dysfunction caused by sepsis, excluding clinical or laboratory evidence of a central nervous system infection, a structural abnormality, or another encephalopathy (such as hepatic encephalopathy or uraemic encephalopathy); SAE refers to a diffuse brain dysfunction resulting from sepsis and is mainly exhibited as delirium, cognitive impairment, decreased learning and memory ability, coma, twitch and so on [3, 6]. The mechanism may involve the dysfunction of cerebral microvascular cells, the loss of blood-brain barrier integrity, mitochondria dysfunction, the activation of microglia and astrocytes, and neuronal death [7, 8]. Currently, the diagnostic criteria and potential risk factors for SAE remain incompletely understood, with no reliable means of clinically evaluating sepsis-associated neurological dysfunction [9]. In the present study, subjective evaluations (performed by clinical staff) and objective indicators (GCS < 15, CAM-ICU for delirium) were combined as the inclusion criteria of SAE patients, and we applied comprehensive exclusion criteria to exclude disturbances due to central nervous system infection, cerebrovascular disease, metabolic disease, sedative-related cognitive effects, toxicosis, etc. More objective indexes should be used in future studies to identify SAE accurately, including craniocerebral ultrasound and bedside electroencephalogram (EEG) monitoring, to detect SAE as early as possible by evaluating cerebral blood flow velocity and changes in EEG activity, especially to predict delirium in critical sepsis patients [4]. The typical changes of EEG in SAE patients include an excessive θ rhythm, an increased δ rhythm, a three-phase wave and burst suppression [10]. In addition, SAE patients exhibit increased brain injury biomarkers (i.e., neuron-specific enolase, S-100 beta-protein) and neuroradiological abnormalities [11–13].

In recent decades, sepsis and SAE have been the focus of intensive medicine research, with high morbidity and mortality. The recently reported 28-day mortality and 180-day mortality rates of SAE were 45.95 and 55.41%, respectively [2]. In the present study, 43.6% of patients with sepsis were identified as having SAE, exhibiting a 42.5% of 28-day mortality, which was significantly higher than that of non-SAE patients. However, there was no significant difference in the hospitalization time, suggesting that encephalopathy resulting from sepsis may have a greater influence on long-term outcomes, including long-term cognitive dysfunction and functional disability, which seriously decrease the quality of life and bring a great burden to patients, their families and even society [14]. In addition, it should be noted that the choice of hospital LOS as an outcome indicator may result in statistical bias. The shortening of LOS may be due to the critical condition of patients and who succumbed during early stages/initial phase of resuscitation, rather than the result of effective treatment.

Elderly patients exhibit a higher risk and mortality from sepsis [15]. In the present study, we also found the age was an independent risk factor for 28-day mortality of SAE patients. In addition, severe patients with underlying diseases often progress more rapidly and have poor prognosis. Especially when the underlying diseases are hypertension, diabetes, acute renal injury, and chronic obstructive pulmonary disease (COPD), such patients might be more likely to develop central nervous system complications [2, 16–18]. We found that patients with sepsis who had underlying hypertension had a higher risk of progression to SAE. Moreover, we found that the SOFA score and APACHE II score were not only independent risk factors for SAE in sepsis patients but also independent risk factors for 28-day mortality in SAE patients. This further confirms the advantages of SOFA scores and APACHE II scores in assessing the severity and prognosis of critical patients [19, 20] and suggests that the underlying health status and severity of sepsis patients are closely related to the occurrence of encephalopathy and adverse outcomes. A significantly decreased platelet count in SAE patients was also found in the present study, which suggested that platelets participate in immune and inflammatory responses against various pathogens, apart from their important role in the coagulation process [21].

It is interesting in the present study that the incidence of gastrointestinal infection and the rate of detection of *Enterococcus* were increased significantly in sepsis patients with encephalopathy, which suggests that the gut or gut microbiota might participate in the pathogenesis of SAE. The reported studies have shown that changes in gut homeostasis and the gut microbiome could influence the physiology, behaviour and cognitive function related to the brain, and the interaction between gut microbiota and the brain has gradually become a hot topic in neuroscience; this interaction may ensure the maintenance of gut homeostasis but also exhibit multiple effects on emotion, motivation and cognitive function [22, 23]. This complex interaction between the gut and brain has been named the “gut-brain axis (GBA)”, which plays an important role in monitoring and integrating intestinal function and linking the emotional and cognitive centres of the brain with peripheral intestinal functions and mechanisms [24]. It has been confirmed that the gut microbiome is involved in the pathophysiological development process of neurodegenerative diseases, stroke, emotional and affective disorders and other central nervous system diseases or encephalopathy [25–27]. Patients with sepsis that is directly influenced by gastrointestinal infection or antibiotic administration [28] may develop disturbances of the gut microbiota. Animal experiments have found that faecal bacteria transplantation can change the gut microbiota of septic mice, suggesting that faecal bacteria transplantation and vagal nerve block are potential therapeutic targets in SAE [29]. The effects of gut microbiota disturbance and the GBA on SAE development deserve further study, especially with respect to upstream and downstream initiation mechanisms, which may provide more ideas to explore the pathogenesis and early diagnosis of SAE.

### Limitations

In summary, in the present retrospective study, we obtained some results that provide certain guiding significance for clinical practice. However, there are still some limitations. First, the relatively small sample size of this single-centre retrospective study and the lower freedom of statistical processing could have impaired the statistical analysis. In addition, we mainly relied on subjective evaluation and exclusive diagnosis to identify SAE. Future studies should add more objective indexes, such as craniocerebral ultrasound and bedside EEG monitoring, to help us identify SAE more accurately and in a timely manner.

## Conclusion

In the present retrospective study, we found that nearly half of patients with sepsis developed SAE, which was closely related to poor outcomes. The identified risk factors for SAE included older age, underlying hypertension, gastrointestinal infection, enterococcal infection, and decreased platelet count. Both the SOFA score and APACHE II score were independent risk factors for predicting the occurrence and adverse outcome of SAE. Future studies should be focused on investigating the long-term prognosis and cognitive function of SAE patients and the upstream initiation mechanism of SAE.

## Supplementary information

**Additional file 1.**

## Data Availability

The datasets used and/or analysed during the current study are available from the corresponding author on reasonable request.

## Abbreviations

SAE: Sepsis-associated encephalopathy
SOFA: Sequential Organ Failure Assessment
APACHE II: Acute Physiology and Chronic Health Evaluation II
GCS: Glasgow coma score
CAM-ICU: Confusion Assessment Method for the Intensive Care Unit
ICU: Intensive Care Unit
WBC: White blood cell
PLT: Platelet
HCT: Haematocrit
PCT: Procalcitonin
Cr: Serum creatinine
LOS: Length of stay
COPD: Chronic obstructive pulmonary disease
GBA: Gut-brain axis
EEG: Electroencephalogram.
