# Supplementary figures and images for: A retrospective study of sepsis-associated encephalopathy: epidemiology, clinical features and adverse outcomes
Source: BMC Emerg Med. 2020 Oct 6;20:77. doi: 10.1186/s12873-020-00374-3 (PMC7539509; doi:10.1186/s12873-020-00374-3)

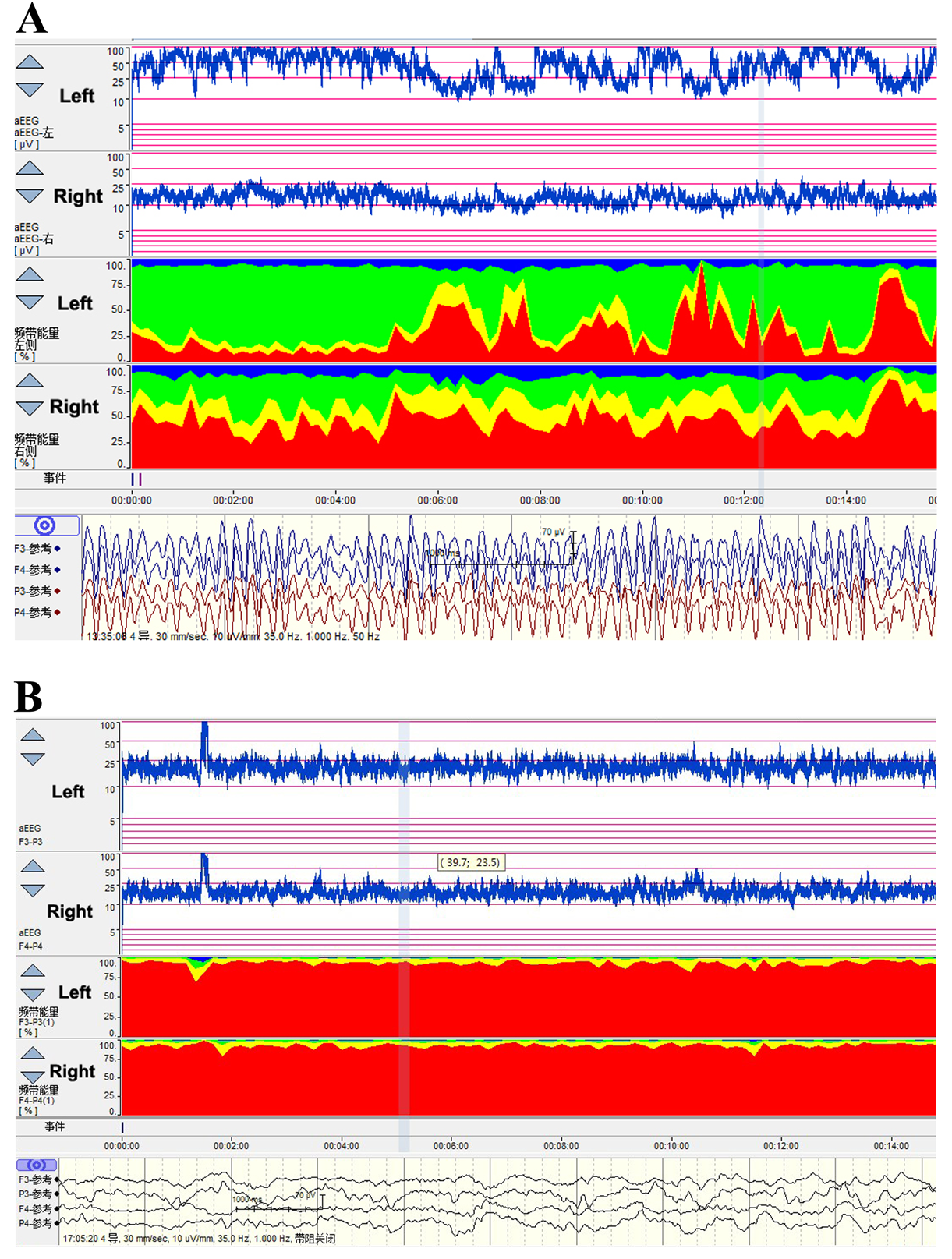

Supplement: Supplementary file 1 — Additional file 1. [file 12873_2020_374_MOESM1_ESM.tif]
